# Exploring the Volatiles Released from Roots of Wild and Domesticated Tomato Plants under Insect Attack

**DOI:** 10.3390/molecules27051612

**Published:** 2022-02-28

**Authors:** Ana Shein Lee Díaz, Muhammad Syamsu Rizaludin, Hans Zweers, Jos M. Raaijmakers, Paolina Garbeva

**Affiliations:** 1 Department of Microbial Ecology, Netherlands Institute of Ecology (NIOO-KNAW), Droevendaalsesteeg 10, 6708 PB Wageningen, The Netherlands; h.zweers@nioo.knaw.nl (H.Z.); j.raaijmakers@nioo.knaw.nl (J.M.R.); p.garbeva@nioo.knaw.nl (P.G.); 2 Institute of Biology, Leiden University, 2333 BE Leiden, The Netherlands

**Keywords:** root volatiles, insect herbivory, PDMS, HiSorb, tomato domestication, *Solanum lycopersicum*, *Solanum pimpinellifolium*, induced defenses, monoterpene, benzyl alcohol, methyl salicylate

## Abstract

Plants produce volatile organic compounds that are important in communication and defense. While studies have largely focused on volatiles emitted from aboveground plant parts upon exposure to biotic or abiotic stresses, volatile emissions from roots upon aboveground stress are less studied. Here, we investigated if tomato plants under insect herbivore attack exhibited a different root volatilome than non-stressed plants, and whether this was influenced by the plant’s genetic background. To this end, we analyzed one domesticated and one wild tomato species, i.e., *Solanum lycopersicum cv* Moneymaker and *Solanum pimpinellifolium,* respectively, exposed to leaf herbivory by the insect *Spodoptera exigua.* Root volatiles were trapped with two sorbent materials, HiSorb and PDMS, at 24 h after exposure to insect stress. Our results revealed that differences in root volatilome were species-, stress-, and material-dependent. Upon leaf herbivory, the domesticated and wild tomato species showed different root volatile profiles. The wild species presented the largest change in root volatile compounds with an overall reduction in monoterpene emission under stress. Similarly, the domesticated species presented a slight reduction in monoterpene emission and an increased production of fatty-acid-derived volatiles under stress. Volatile profiles differed between the two sorbent materials, and both were required to obtain a more comprehensive characterization of the root volatilome. Collectively, these results provide a strong basis to further unravel the impact of herbivory stress on systemic volatile emissions.

## 1. Introduction

Plant volatiles have been extensively studied due to their wide range of chemical classes and ecological functions [1]. Furthermore, the plant volatilome is considered as an extended metabolome, reflecting the plant’s physiological status. Often, the term volatilome refers to the totality of volatile compounds emitted by an organism under specific conditions [2]. Of particular ecological relevance are the volatile organic compounds (VOCs), small molecules with low molecular weight (<300 Da), lipophilic character and high-vapor pressure. VOCs emitted by a plant constitute a wide range of chemical classes including terpenes, terpenoids, alcohols, carbonyl compounds, aliphatic hydrocarbons, aromatic, sulfur and nitrogen containing compounds [3]. The rich chemical diversity of the plant’s volatilome is of ecological relevance, in particular for chemical communication with other (micro)organisms. For instance, plants emit volatiles to indicate the presence of open flowers, attack by herbivores, production of ripe fruit, and pathogen infection [4]. The emission of plant volatiles can significantly differ under non-stressed conditions (constitutive emission) or stressed conditions (induced emission). For example, upon fungal infection, the roots of *Carex arenaria* emitted different terpenes (e.g., the monoterpene (Z)-limonene oxide than roots of noninfected plants [5]. Plants are constantly challenged by different biotic and abiotic stresses and the emission of induced volatiles can directly reduce the intensity of the stress or act as indirect defense by attracting natural enemies (predators, parasitoids) of insect herbivores [6]. However, chemical-ecological studies have focused mostly on plant volatiles in aboveground interactions, whereas the chemical diversity and importance of volatiles in belowground communication has received much less attention.

Similar to aboveground plant tissues, stress-related responses involving secondary metabolites occur belowground. Compared to soluble compounds that accumulate around the root epidermal cells, volatiles can readily diffuse via air- and gas-filled pores in the soil matrix and play a role in long-distance interactions [7]. A recent study in our lab revealed differences in root-emitted volatiles between roots of healthy tomato plants and those infected by the root pathogenic fungus *Fusarium oxysporum* [8]. Root volatiles are not only produced locally upon infection by root pathogens but can also be induced systemically belowground upon stress aboveground [9]. For example, in Brassicaceae plants, roots release sulfurous volatiles in response to aboveground herbivory [10]. Plant volatile production upon stress is regulated by plant hormones, with jasmonic acid (JA) as a key hormone in plant defense against insect herbivores and in the production of terpenes [11]. Upon local or systemic stress, root volatiles can play multiple roles in indirect defense, serving as chemoattractant or as a carbon source for root-associated beneficial microbes [12].

The trade-off between constitutive and induced plant defenses has been proposed to be affected by plant domestication [13]. For example, several modern tomato cultivars have less tolerance to insect pests than their wild relatives [14]. Contrarily, it has been observed that the modern species *S. lycopersicum cv* Better Boy has a higher ability of induced defenses compared to wild tomato species, and increased volatile production upon herbivory stress [15]. Nonetheless, the relationship between domestication and root volatile emissions remains largely elusive. 

The aim of our study is to explore the root volatilome of a wild and a modern tomato species under herbivory stress. We trapped the root volatiles from the headspace in a compartmentalized setup using two different sorbent materials: polydimethylsiloxane (PDMS) in silicon-based tubes and commercial coated probes (HiSorb^TM^). These materials have been reported to be widely used for passive sampling of volatiles from soil and roots [16,17,18]. Combining these two trapping methods, we compared the root volatilome of the domesticated tomato *Solanum lycopersicum cv* Moneymaker with that of its wild relative *Solanum pimpinellifolium* under attack of the leaf herbivore insect *Spodoptera exigua*. Our hypothesis is that upon the same stress, the two tomato species have a different capacity of mounting defenses that is reflected in a different root volatile profile.

## 2. Results

We investigated the root volatilome of the two tomato species *S. pimpinellifolium* and *S. lycopersicum cv* Moneymaker (hereafter referred to as *S. lycopersicum*) under stress in an in vitro setup (Figure S1). For that purpose, five replicates (*n* = 5) of each species were subjected to leaf-chewing herbivore insect *S. exigua*. (*S. exigua* = SE). Two trapping materials, PDMS and HiSorb, were placed in the root headspace compartment 24 h after insect exposure. Similarly, five replicates of each species without insect pest were used as non-stressed control plants (Control *S. exigua* = CSE).

### 2.1. Root Volatile Compound Detection Is Trapping-Material Dependent

**Figure 1:**
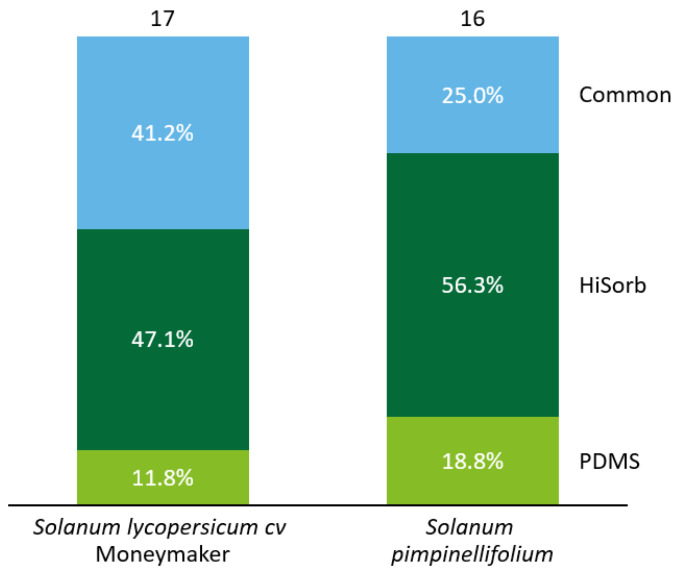
Percentage of volatile compounds detected from roots of wild (*S. pimpinellifolium*) and domesticated (*S. lycopersicum*) tomato by two different trapping materials (HiSorb and PDMS). From top to bottom: total number of volatile compounds detected, percentage of volatile compounds detected commonly by both trapping materials (Common), exclusively detected by HiSorb or exclusively detected by PDMS.

By employing two trapping materials (HiSorb and PDMS), a total of 17 volatile compounds were detected in the *S.* *lycopersicum* root headspace and 16 compounds in that of *S. pimpinellifolium* (Figure 1). More volatile compounds were detected with HiSorb than with PDMS traps in both tomato species. From the 17 compounds detected in *S. lycopersicum*, seven compounds (41.2%) were commonly detected by HiSorb and PDMS. The remaining ten compounds were detected either with HiSorb (eight) or PDMS (two). In a similar way, for *S.* *pimpinellifolium,* the two trapping materials commonly detected only four (25%) out of the total compounds (Figure 1). The majority of the remaining volatile compounds were detected by HiSorb (nine) in comparison to PDMS (three). In conclusion, the detection of root-volatile compounds is highly trapping-material dependent.

**Figure 2:**
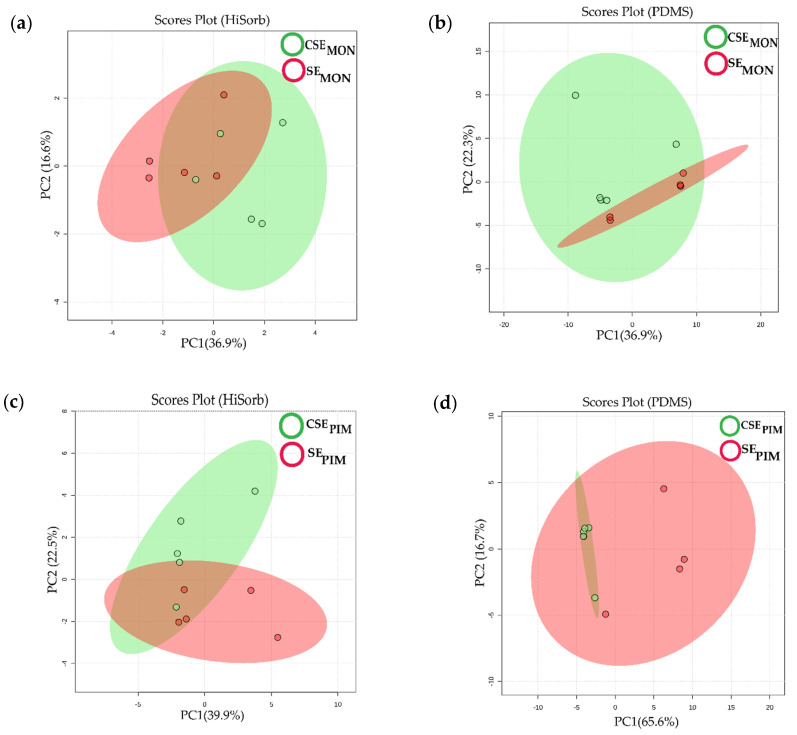
Differences in root volatilome profiles between stressed (SE-red) and control (CSE-green) upon 24 h leaf hebivory. Principle component (PC) plots of *S. lycopersicum* root volatilomes trapped with HiSorb (**a**) or PDMS (**b**) and of *S. pimpinellifolium* trapped with HiSorb (**c**) or PDMS (**d**). For each plot, a single coloured dot represents the volatilome of one sample. Distances between dots are equivalent to variation between replicates. The shadowed coloured cluster represents the 95% interval of confidence.

The differences between trapping materials, therefore, did not only affect the number of identified volatile compounds, but also the volatilome of each tomato species exposed (or not) to insect stress. The volatilome of each sample consists of the number of volatile compounds and their relative concentration or intensity in the volatile profile. The principal component analysis (PCA) showed that the root volatilome of control (CSE) and stressed (SE) plants differed between species (Figure 2) and these differences were also affected by the trapping materials. Regardless of the species, the root volatilome detected by HiSorb presented a clearer separation between CSE and SE than the volatilome detected by PDMS (Figure 2a–d).

### 2.2. Effects of Leaf Herbivory on Root Volatilome

**Figure 3:**
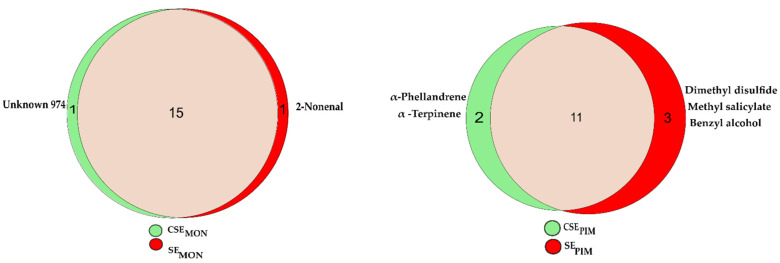
Qualitative overview of root volatiles present uniquely in stressed (SE), control plants (CSE) or in both. Each Euler plot (left *S. lycopersicum* Moneymaker (MON), right *S.* *pimpinellifolium* (PIM)) shows the stress-related compounds in red as SE_MON_ and SE_PIM_ respectively, in the beige intersection the common compound found in both treatments and in green as CSE_MON_ and CSE_PIM_ respectively the nonstress related compounds.

To obtain a more comprehensive view of the root volatilome, we analyzed the combination of compounds detected by both trapping materials for qualitative analysis. This analysis aimed to find differences in the type of volatile compounds induced by insect herbivory on *S. lycopersicum* and on *S.* *pimpinellifolium. S.* *pimpinellifolium* presented the largest difference between control (CSE) and insect stress (SE). Stress-related compounds were those present in three or more replicates in stressed (SE) plants and less than three replicates in control (CSE) plants. Following these criteria, 2-nonenal was identified as a stress-related compound in *S. lycopersicum,* whereas dimethyl disulfide (DMDS), methyl salicylate (MESA), and benzyl alcohol were identified as stress-related compounds in *S. pimpinellifolium* (Figure 3). Contrarily, some compounds were not found in stressed plants but only in control plants, thus were considered as nonstress-related compounds. Those were the unknown 974 in *S. lycopersicum* and α-phellandrene and α-terpinene in *S.* *pimpinellifolium* (Figure 3).

Despite their different volatilomes, the wild and modern tomato species shared ten common volatile compounds: a group of six terpenes (α-phellandrene, α-pinene, α-terpinene, β-phellandrene, D-limonene, and p-cymene), benzyl alcohol, methyl salicylate and the unknown compounds Unknown 1087 and Unknown 1114 (Table 1 and Table 2). 

Considering the two trapping materials, 17 compounds were identified in the *S. lycopersicum* root headspace (Table 1). These compounds represented six different chemical classes: seven monoterpenes (α-pinene, 2-carene, α-phellandrene, α-terpinene, p-cymene, d-limonene, and β-phellandrene), two oxygenated aliphatic compounds (heptanal, 2-nonenal), an aliphatic hydrocarbon (3-nonene), an alcohol (benzyl alcohol), an aromatic ketone (1-(2-hydroxyphenyl)-ethanone) and an aromatic ester (methyl salicylate). Most compounds were commonly identified in both treatments; only 2-nonenal was considered as a stress-related compound. Also, benzyl alcohol and unknown 1172 were considered as stress-related compounds but only according to one trapping material (Hisorb and PDMS, respectively) (Table 1). 

**Figure 4:**
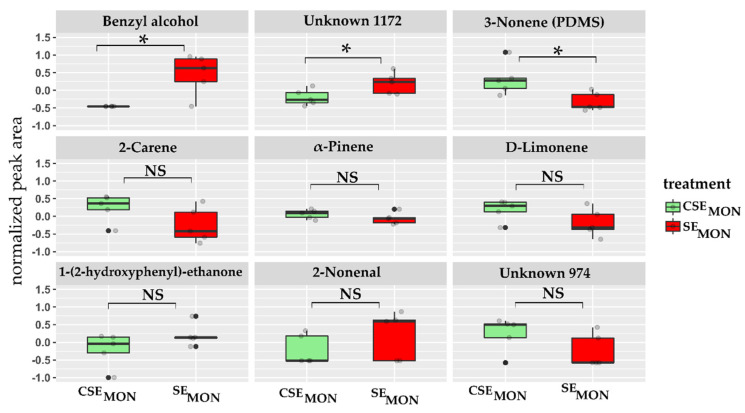
Peak areas of main compounds detected in control CSE_MON_ (green) and herbivory-stressed SE_MON_ (red) *S. lycopersicum* roots. All compounds were detected with HiSorb except for 3-nonene (PDMS). Each box-plot depicts the distribution and median (horizontal line) of normalized and log10 transformed peak area of five replicates per treatment. oxplots indicated with an asterisk (*) differ statistically (*p* < 0.05), whereas box-plots indicated with NS did not show statistically significant differences.

We performed a quantitative analysis to determine differences in compound intensity upon stress. Univariate analysis (T-test) confirmed that in *S. lycopersicum*, three compounds presented a significantly different peak area between control and stressed plants. Benzyl alcohol and unknown 1172 presented an increased emission in SE_MON_ plants (*p*-value: benzyl alcohol = 0.008 *, unknown 1172 = 0.046 *), whereas 3-nonene was higher in CSE_MON_ plants (*p*-value 3-nonene = 0.026 *) (Figure 4). Although there was no statistical significance, a decrease in terpene production under stress conditions was observed; e.g., 2-carene, D-limonene, and α-pinene, present in both treatments, showed higher peak areas in CSE_MON_ plants. Similarly, 1-(2-hydroxyphenyl)-ethanone peak area was also reduced upon stress. Despite 2-nonenal was only detected in stressed SE_MON_ plants, it did not present statistical differences in peak area between treatments (Figure 4).

In *S. pimpinellifolium* plants, 16 compounds were detected considering both trapping materials (Table 2). The compounds represented four chemical classes: monoterpenes (α-pinene, camphene, β-pinene, α-phellandrene, α-terpinene, p-cymene, D-limonene, β-phellandrene, and γ-terpinene), one sulfur compound (DMDS), an aromatic ester (MESA), and an alcohol (benzyl alcohol). Despite representing fewer chemical classes than its modern relative *S. lycopersicum*, this species showed more qualitative differences between stressed and non-stressed volatilomes by emitting three stress-related compounds and three nonstress-related compounds (β-phellandrene only when detected with HiSorb).

**Figure 5:**
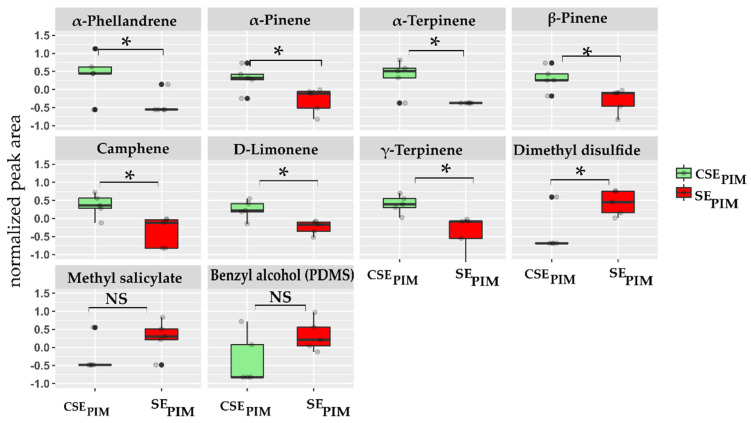
Peak areas of compounds detected by HiSorb in control CSE_PIM_ (green) and herbivory-stressed SE_PIM_ (red) *S. pimpinellifolium* roots. Each box-plot depicts the distribution and median (horizontal line) of normalized and log10 transformed peak areas of five replicates per treatment. Boxplots indicated with an asterisk (*) differ statistically (*p* < 0.05), whereas box-plots indicated with NS did not show statistically significant differences

From a quantitative perspective, eight compounds presented a significantly different peak area between control and stressed plants (Figure 5). Univariate analysis (T-test) confirmed that in *S. pimpinellifolium*, seven terpene compounds presented a significantly higher peak area in control CSE_PIM_ plants (*p*-values: α-terpinene = 0.006 **, D-limonene = 0.01 **, camphene = 0.016 *, γ-terpinene = 0.016 *, β-pinene = 0.023 *, α-phellandrene = 0.027 * and α-pinene = 0.027 *). Hence, upon leaf-herbivory stress, a significant reduction of terpene emission was observed in the wild tomato species. In particular, α-phellandrene and α-terpinene were uniquely present in control (as previously shown in Table 2 and Figure 3), whereas α-pinene, β-pinene, camphene, D-limonene, and γ-terpinene were present in both treatments but with different peak areas (normalized peak area). Dimethyl disulfide, benzyl alcohol, and methyl salicylate emissions were increased under stress conditions (SE_PIM_), but only DMDS was statistically significant (*p*-value dimethyl disulfide = 0.02 *).

## 3. Discussion

Plants release a vast array of primary and secondary metabolites from their roots including, volatile organic compounds. However, studies on root volatiles remain challenging due to the complexity of belowground volatile-trapping and sample preparation [20,21]. Factors like the compound volatility, soil pore diameter, composition of the solid soil matrix phase, and the relative humidity of the soil matrix can impact the diffusion and thus detectability of the compounds [7]. Therefore, we developed a compartmental system allowing root-volatile collection in a controlled in vitro design (Supplementary Figure S1). This system enables the passive trapping of the volatiles through a headspace in a sterile, nondestructive and scalable system. By employing this two-compartment system, we also ensure that the compounds trapped are only volatiles released by roots. In addition to the experimental design, the selection and comparison of the trapping materials is often overlooked and rarely included in the experimental setup. Recently, Diez-Simon et al. [22] have shown that measuring the same sample with different trapping methods can provide different volatile profiles; some extracted a broad spectrum of chemical classes, while others presented higher affinity for certain chemical classes. This study exemplifies the importance of including different trapping methods or materials and selecting the most appropriate for the study. 

In our experiments, we compared and combined the trapping capacity of PDMS tubes versus commercial HiSorb probes. In general, each material presented a different trapping capacity with HiSorb traps detecting more compounds than PDMS. The number of commonly detected compounds ranged from 25% to 41% (in *S. pimpinellifolium* and *S. lycopersicum,* respectively) of the overall volatilome (considered here as the sum of volatiles detected by both trapping materials). Thus, if the trapping relies only on one sorbent material, the representativity of the volatilome might be incomplete, leading to possible misinterpretations or biased conclusions. Although HiSorb probes contain the same extraction phase (polydimethylsiloxane) as the PDMS tubes, they showed a higher trapping capacity. It might be possible that the coating of the extraction phase in HiSorb probes improves the sensitivity of the trap, whereas, in PDMS tubes, there might be a greater compound competition for the extraction phase. It is known that volatile competition for the phase is a disadvantage of solid-phase microextraction (SPME) and that the most concentrated compounds can saturate the phase’s surface [21,22,23]. Therefore, despite having the same extraction phase material, HiSorb probes and PDMS tubes can have different volatile concentration equilibria and capacity of compound retention. 

We combined the compounds detected by the two trapping materials to obtain a more comprehensive root volatilome for each species. Both species differed in the number and type of stress-associated compounds. The compound detectability and therefore results interpretation, was affected by the sensitivity of trapping materials. Regardless of the species, PCA analysis showed that volatile profiles detected with HiSorb presented a clearer difference between CSE and SE treatments than those trapped with PDMS. Such differences may be due to the higher efficiency of HiSorb than PDMS.

In general, the wild species *S. pimpinellifolium* presented the largest volatilome differences between control and herbivore-stressed plants; for instance, stressed SE_PIM_ plants emitted the sulfur compound dimethyl disulfide (DMDS). Plant production of DMDS upon systemic damage has been documented [8,10] but to our knowledge, the induced production of DMDS in tomato roots upon aboveground herbivory has not been yet reported. Other stress-related compounds found in *S. pimpinellifolium* were methyl salicylate and benzyl alcohol. Methyl salicylate (MESA) is the methyl ester of the phytohormone salicylic acid (SA), which has a crucial role in plant defense. MESA, a volatile version of SA, might be produced by the plant to control the pool of active SA [24] and as a systemic messenger of defense signals [25]. However, MESA has been related to susceptibility in tomato to *Fusarium oxysporum* f. sp. *lycopersicum* [26] and attraction of root nematodes [27]. Although the exact role of MESA is not fully understood, it is possible that MESA levels can be an indirect marker of plant defense activation. Similar to MESA, benzyl alcohol has also been detected as stress-related in *S. lycopersicum* roots, being the only common stress-related compound between the two species. Benzyl alcohol has been reported as a volatile compound related to drought-stress in tea plants and emitted upon the release of glycosides during cell destruction [28]. The same compound was also emitted by *P. trichocarpa* leaves upon the infestation by the poplar leaf beetle (*Chrysomela populi*) [29]. Recently, a study has associated benzyl alcohol emission with fragrance absence in flowers from apricot trees [30]. Through transcriptomic analysis, the authors found a correlation between the activation of the phenylalanine ammonia-lyase (PAL) enzyme and the conversion of the scented volatile benzyl acetate into benzyl alcohol in nonfragrant flowers. While such volatile was documented aboveground, the evidence for the emission belowground remains elusive. In *S. lycopersicum* plants, in addition to benzyl alcohol, 2-nonenal and the unknown 1172 (possibly an aliphatic hydrocarbon) were also emitted under insect stress conditions. The volatile compound 2-nonenal is derived from fatty acid peroxidation in tomato leaves [31]. This compound has also been reported to impair spore germination of pathogenic fungi [32] and was associated with susceptibility to *Fusarium oxysporum* f. sp. *lycopersicum* [26].

Some volatiles were mainly detected under nonstress conditions (CSE). For both species, we observed an overall reduction of monoterpene production upon stress, which can be related to the trade-off between constitutive and induced defense [33,34]. Particularly, in *S. pimpinellifolium*, seven terpenes were significantly reduced upon stress. The systemic volatile emission is regulated by a complex phytohormonal balance. Studies have shown a positive correlation in leaves for jasmonic acid (JA)-terpene production: e.g., JA-deficient tomato plants were more susceptible to *S. exigua* due to lower production of terpenes [11]. Another study showed that upon *Botrytis cinerea* infection, tomato plants increase the production of terpenes derived from the lipoxygenase pathway (LOX) [35]. However, a root-specific monoterpene synthase was shown to be unaffected by herbivore wounding or JA application [36]. To date, the complex regulation of terpene production in the roots and particularly how it is affected by the aboveground stresses remains unknown. 

The relationship between domestication and plant defense is still largely unknown. However, there is evidence about the link between aboveground stresses and genotype-dependent root volatile production. It has been shown that domestication negatively impacted the emission of the root volatile compound (E)-β-caryophyllene by maize plants, hence their ability to attract natural enemies (entomopathogenic nematodes) of the insect pest *Diabrotica virgifera virgifera* [34,37]. The domestication of tomato plants has been studied in the context of microbial recruitment and community assembly in the rhizosphere [38,39]. However, the impact of domestication on the root metabolome and in particular, on the root volatilome is still understudied. In our simplified in vitro system, we demonstrated that the root volatilome differs between a wild and a domesticated tomato species. It should be emphasized that the number of species tested is too small and should be extended to identify the impact of domestication on root volatilomes. Nevertheless, our survey did reveal that the genetic background of the host plant affects the root volatilome in response to leaf herbivory, both quantitatively and qualitatively. This study paves the way for further functional analyses to unravel the impact of plant domestication on the production of root volatiles under biotic stresses.

## 4. Materials and Methods

### 4.1. Plant Growth and Herbivory Stress Induction

Two tomato species, *Solanum lycopersicum* *cv* Moneymaker (hereafter referred to as *S. lycopersicum*) (purchased from Bingenheimmer Saatgut AG, Echzell, Germany) and *Solanum pimpinellifolium* (provided by Wageningen University, Wageningen, The Netherlands), were used. Tomato seeds were surface sterilized for 2 min in 70% (*v*/*v*) ethanol followed by 15 min in 1.5% (*v*/*v*) aqueous sodium hypochlorite soln. and then washed three times with sterile distilled water. Then, sterile seeds were sown in one side of two-compartment Petri dishes (UV-sterilized) containing 20 mL of 0.5 Murashige and Skoog (MS) medium (1% agar, 1% sucrose, pH 5.7). The other compartment remained empty for volatile trapping (Figure S1). The plates were sealed with parafilm and each set of replicates (*n* = 5) from a treatment was put in an individual large plastic box and incubated in a climate chamber. The incubation condition was set constantly at 23 °C, 16/8 h light/dark photoperiod (180 mmol PAR) until harvest. After reaching 3–4 true leaves, five replicates of each tomato species were stressed by *Spodoptera exigua*. Briefly, *S. exigua* eggs (obtained from Entocare N.V., Netherlands) and hatched larvae were reared with artificial diet (Table S2) in a growth chamber (20 °C) for 10 days prior to their introduction to plants. After the rearing period, two larvae at third-instar stage (L3) were put inside a mesh bag that covered each plant replicate (SE) for 24 h (Figure S1). Control plants were covered with the mesh bag but did not contain any caterpillar (CSE).

### 4.2. Trap conditioning (PDMS and HiSorb)

Polydimethylsiloxane (PDMS) tubes (internal diameter 1 mm, external diameter 1.8 mm, Carl Roth, Karlsruhe, Germany) were cut into pieces with a length of 5 mm. For each sample, two pieces were inserted in a sterile needle for easy handling. For conditioning, the tubes were fully immersed on a mixture acetonitrile/methanol (4/1, *v*/*v*) and incubated for 16 h (overnight) at room temperature. The tubes were then dried under pure nitrogen (N2) flow (5 L min^−1^) and heated at 210 °C for 1.5 h under helium flow (5 L min^−1^). The conditioned tubes along with the needles were then stored in clean glass vials (previously flushed with argon for 10 s). For conditioning the HiSorb material (model H1-AXABC, Markes International Ltd., Llantrisant, UK), probes were preconditioned at 280 °C for 1 h using a U-CTE micro-chamber/thermal extractor (Markes International Ltd., Llantrisant, UK) prior to insertion into a clean, empty metal holder with screw caps at both ends (Markes International Ltd., Llantrisant, UK). The metal holders containing HiSorb probe were then stored in a well-ventilated laboratory at room temperature until usage.

### 4.3. Root Volatile Collection

The in vitro cultivation of tomato plants using sterile two-compartment Petri dishes allows passive collection of root volatiles while minimizing interfering volatiles originating from contaminants. Passive trapping was done by introducing HiSorb and PDMS traps in the empty side of the two-compartment Petri dish. All compounds trapped in the root headspace with a passive diffusion method were considered volatiles. To maintain system in sterility, the plates were opened under the flow cabinet and subsequently the traps were placed into each empty compartment. For plants from experiment two (*S. exigua* infested) root volatiles of five replicates (SE *n* = 5, CSE *n* = 5) were collected simultaneously by one HiSorb and two PDMS tubes per plate after 24 h of stress introduction. Additionally, for the negative control, the traps were also placed in the empty side of the two-compartment plates containing only MS medium (without any plants). Plates were sealed again with parafilm and the traps stayed inside for approximately 3 h at the same conditions as the growth chamber (20 °C). Then, the plates were brought back again under the flow cabinet to extract the traps and stored them until measurement (HiSorb probes at room temperature and PDMS tubes in vials at −20 °C).

### 4.4. GC/Q-TOF Measurement

The volatile organic compounds were desorbed from PDMS tubes and from HiSorb probes using an automatic desorption unit (Unity TD-100, Markes International, Llantrisant, UK) with the helium gas at 50 mL min^−1^ at the temperature of 240 °C for 8 min. The released volatile compounds were then trapped with a cold trap at −10 °C and reheated at 280 °C for 5 min. The volatiles were then transferred splitless or with split 1:9 (280 °C transfer line) to the GC/Q-TOF (model Agilent 7890B GC and the Agilent 7200A Q-TOF, Santa Clara, CA, USA) with an DB-5 ms ultra-inert column (30 m length, 0.25 mm internal diameter, 0.25 μm film thickness, Agilent Technologies, Inc., Santa Clara, CA, United States) with a run time of 35.6 min and a flow of 1.2 mL/min (constant flow). The temperature program was set to 39 °C for 1 min followed by heating up to 315 °C with 10 °C/min and holding for 7 min. Then, volatile compounds were detected by the GC/Q-TOF system running at 70 eV in electron ionization (EI) mode with a temperature source of 230 °C. The mass spectra of the volatile compounds were acquired in full-scan-mode (*m*/*z* 30–400, 4 scans/s, 2 GHz Extended Dynamic Range). Calibration of retention index (RI) was calculated from a reference alkane standard solution; 1μl was injected in an empty Tenax trap and measured with the same parameters as described before.

### 4.5. GC/Q-TOF Data Analysis

GC/Q-TOF raw data (.D) were translated to content definition file (.cdf) format with GC AIA translator B.07.04 (Agilent Technologies, Santa Clara, CA, USA). Converted files were then imported into MzMine v2.53 [40,41] for further analysis. Briefly, Automated Data Analysis Pipeline (ADAP) algorithm was employed to perform chromatogram building, peak deconvolution, spectral deconvolution and alignment steps [42] (parameters details are provided in Table S2. The peak intensity (area) tables were then exported as comma-separated value (.csv) files. Subsequently, the mass features were manually checked and only selected for qualitative analysis when present at least in 3 out of 5 replicates to be considered as true features representing volatile compounds. Furthermore, mass features were further filtered by eliminating those that were also present in MS medium treatment, to ensure that the compounds are only emitted by roots. Volatile identification was performed using Mass Hunter (Qualitative Analysis v10, Agilent Technologies Inc.) by comparing their retention index (RI) and mass spectrum with the NIST 2020 V2.20 (National Institute of Standards and Technology, United States) which consists of more than 300,000 spectra representing more than 300,000 unique compounds. Further details on the mass spectra comparison are available in Supplementary information. Compounds with a RI differences larger than 10 index units were not considered [43]. Furthermore, compound identification was based on the metabolite identification categories described by Sumner et al. [19]: unknown compounds = level 4 (quantified and differentiated by spectral data), rest of compounds = level 2 (putatively annotated based on spectral similarity to public database). Briefly, we assigned a compound name when it matched to a mass spectrum and retention index (RI) of the NIST library, otherwise we described it as “unknown” for which identification level was not conclusive. 

### 4.6. GC/Q-TOF Data Visualization and Statistical Analysis

To elaborate the graphs, we combined the compounds detected in both HiSorb and PDMS to obtain a comprehensive volatile list. The Euler plots were elaborated by assessing compound presence or absence based on a minimum detection level of 3 out of 5 replicates using R studio (R Studio 2021.09.0, build 351). The principal component analysis (PCA) plots were based on the peak intensity (area) tables of filtered mass features (minimum *n* = 3/5 replicates) obtained as explained in Section 4.5 and made with MetaboAnalyst v 5.0 [44]. For statistical analyses, the same peak area tables of the filtered mass features were normalized via logarithmic transformation and mean-centering using the MetaboAnalyst R package [44]. *t*-test was applied to analyze peak area differences between treatments.

## 5. Conclusions

Our study shows that the root volatilome of tomato can be impacted by domestication. The wild relative *Solanum pimpinellifolium* presented a different volatilome than its modern relative, *Solanum lycopersicum cv* Moneymaker. In addition, the two species reacted differently to herbivory stress, both quantitatively and qualitatively. The wild species revealed the largest differences between stressed and non-stressed volatilome in terms of the number of induced compounds detected and loss of or reduced emission of constitutively emitted compounds. Characterization of the root volatilome of the two species, both under stress and non-stressed condition, was trapping-material dependent. In particular, HiSorb was the sorbent material that presented the best trapping capacity across samples and treatments. Nonetheless, neither of the two trapping materials fully trapped the totality of the compounds detected. Thus, in order to improve the accuracy of volatile detection, the selection of the trapping material and use of more than one trapping material are crucial for reliable volatilome analyses and unbiased ecological conclusions about the role of volatiles.

## Figures and Tables

**Table 1:** List of root volatile compounds identified in *S. lycopersicum cv* Moneymaker in stressed (SE_MON_) and non-stressed (CSE_MON_) plants detected with PDMS and HiSorb. Compounds marked with an asterisk (*) in the Treatment column were uniquely detected in the stressed (SE) or non-stressed (CSE) treatments with the indicated trapping material.

Compound ^1^	RI ^2^	RI Database	Treatment		Sorbent Material		Molecular Formula	CAS
*Solanum lycopersicum cv* Moneymaker			CSE_MON_	SE_MON_	PDMS	HiSorb		
3-Nonene	892	896	x	x	x		C_9_H_18_	20063-77-8
Heptanal	903	901	x	x	x		C_7_H_14_O	111-71-7
α-Pinene	936	937	x	x		x	C_10_H_16_	80-56-8
Unknown 974	974	NA	x *			x	NA	NA
2-Carene	1001	1002	x	x	x	x	C_10_H_16_	554-61-0
α-Phellandrene	1009	1005	x	x		x	C_10_H_16_	99-83-2
α-Terpinene	1019	1017	x	x		x	C_10_H_16_	99-86-5
P-Cymene	1028	1025	x	x	x	x	C_10_H_14_	99-87-6
D-Limonene	1032	1031	x	x	x	x	C_10_H_16_	5989-27-5
β-Phellandrene	1034	1031	x	x	x	x	C_10_H_16_	555-10-2
Benzyl alcohol	1038	1036	x	x *	x	x *	C_7_H_8_O	100-51-6
Unknown 1087	1087	NA	x	x	x	x	NA	NA
Unknown 1114	1114	NA	x	x		x	NA	NA
2-Nonenal	1161	1162		x *		x	C_9_H_16_O	18829-56-6
1-(2-Hydroxyphenyl)-ethanone	1166	1167	x	x		x	C_8_H_8_O_2_	118-93-4
Unknown 1172	1172	NA	x	x *	x *	x	NA	NA
Methyl salycilate	1199	1192	x	x		x	C_8_H_8_O_3_	119-36-8

^1^ Identification of compounds based on the metabolite identification categories described by Sumner et al. [19]. List of mass spectrum match profiles with NIST 2020 database provided in Supplementary information. ^2^ RI is the retention index calculated from retention time and a reference n-alkane mix: RI database is the reference retention index used for identification from NIST 2020 database.

**Table 2:** List of root volatile compounds found in *S. pimpinellifolium* in stressed (SE_PIM_) and non-stressed (CSE_PIM_) plants detected with PDMS and HiSorb. Compounds marked with an asterisk (*) in the Treatment column were uniquely detected in the stressed (SE) or non-stressed (CSE) treatments with the indicated trapping material.

Compound ^1^	RI ^2^	RI Database	Treatment		Sorbent Material		Molecular Formula	CAS
*Solanum pimpinellifolium*			CSE_PIM_	SE_PIM_	PDMS	HiSorb		
Dimethyl disulfide	747	746		x *		x	C_2_H_6_S_2_	624-92-0
α-Pinene	936	937	x	x	x	x	C_10_H_16_	80-56-8
Camphene	953	952	x	x		x	C_10_H_16_	79-92-5
β-Pinene	981	979	x	x		x	C_10_H_16_	127-91-3
α-Phellandrene	1009	1005	x *			x	C_10_H_16_	99-83-2
Unknown (1011)	1011	NA	x	x		x	NA	NA
α -Terpinene	1019	1017	x *			x	C_10_H_16_	99-86-5
P-Cymene	1028	1025	x	x	x	x	C_10_H_14_	99-87-6
D-Limonene	1032	1031	x	x	x	x	C_10_H_16_	5989-27-5
β-Phellandrene	1034	1031	x *	x	x	x *	C_10_H_16_	555-10-2
Benzyl alcohol	1038	1036		x *	x		C_7_H_8_O	100-51-6
γ-Terpinene	1061	1060	x	x		x	C_10_H_16_	99-85-4
Unknown (1087)	1087	NA	x	x	x		NA	NA
Unknown (1114)	1114	NA	x	x	x		NA	NA
Unknown (1166)	1166	NA	x	x		x	NA	NA
Methyl salicylate	1199	1192		x *		x	C_8_H_8_O_3_	119-36-8

^1^ Identification of compounds based on the metabolite identification categories described by Sumner et al. [19]. List of mass spectrum match profiles with NIST 2020 database is provided in Supplementary information. ^2^ RI is the retention index calculated from retention time and a reference n-alkane mix: RI database is the reference retention index used for identification from NIST 2020 database.

## Data Availability

Raw data files (in .cdf fomat) can be found in the Zenodo database accession number 5788930 (https://doi.org/10.5281/zenodo.5788930).

## Supplementary Materials

The following are available online. Figure S1: Schematic representation of the in vitro experimental set up of experiment; List of mass spectra of identified compounds; Table S1: *Spodoptera exigua* artificial diet; Table S2: Parameters used for data processing using MZmine 2.53 (ADAP); Figure S2: Comparison of root biomass (dry weight) between treatments; Figure S3: Correlation plot between root VOC (α-pinene) with root biomass.
