# Supplementary material for: Exploring the Volatiles Released from Roots of Wild and Domesticated Tomato Plants under Insect Attack
Source: Molecules. 2022 Feb 28;27(5):1612. doi: 10.3390/molecules27051612 (PMC8911868; doi:10.3390/molecules27051612)

Exploring the Volatiles Released from Roots of Wild and Domesticated Tomato Plants under Insect Attack

Ana Shein Lee Díaz [1,*,†], Muhammad Syamsu Rizaludin [1,*,†], Hans Zweers [1], Jos M. Raaijmakers [1,2] and Paolina Garbeva [1]

Supplementary Information

1. **FigureS1.** Schematic representation of the in vitro experimental set up
2. **List of mass spectra of identified compounds**
3. **Table S1.** *Spodoptera exigua* artificial diet
4. **Table S2.** Parameters used for data processing using MZmine 2.53 (ADAP)
5. **FigureS2.** Comparison of root biomass (dry weight) between treatments
6. **FigureS3.** Correlation plot between root VOC (α-pinene) with root biomass

FigureS1. Schematic representation of the in vitro experimental set up. Plants growing on 0.5MS media on the right side and in the left empty compartment one HiSorb and two PDMS tubes. Control plants CSE are covered with a mesh bag but do not contain *Spodoptera exigua*. *Spodoptera exigua*-stressed plants (SE) contain a mesh bag with two caterpillars for 24h**.**

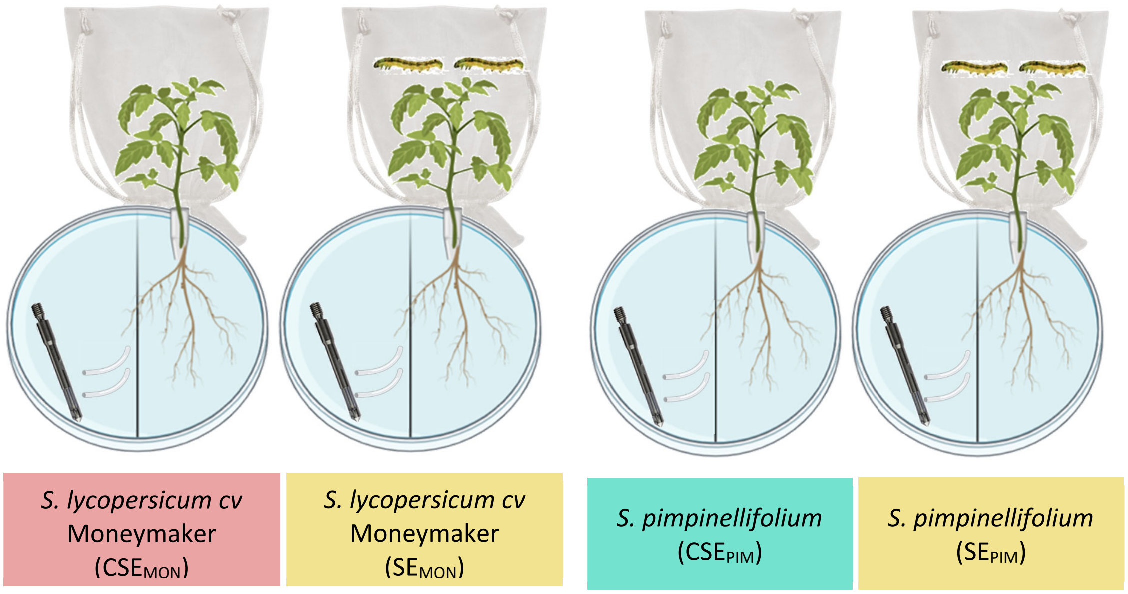

2. List of mass spectra of identified compounds

List of identified compounds using mass spectrum match with NIST 2020 library (in RT/RI ascending order). Plot of mass spectra pairwise comparison between sample (red) and library (blue) hit.

RT 3.3 min / RI 747 / Dimethyl disulfide

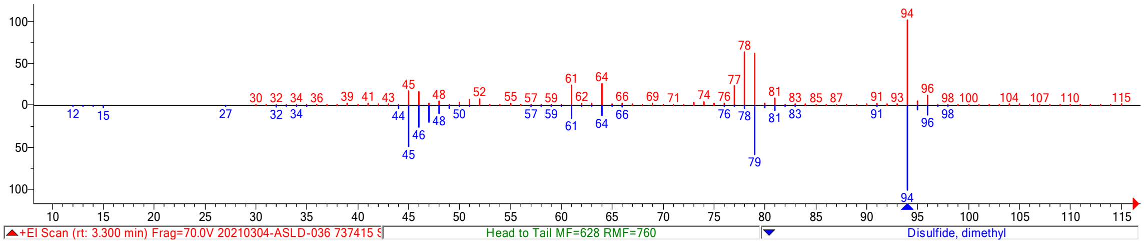

Name: Disulfide, dimethyl
Formula: $C_2H_6S_2$
MW: 94 Exact Mass: 93.991092 CAS#: 624-92-0 NIST#: 291515 ID#: 83620 DB: mainlib
Other DBs: Fine, TSCA, RTECS, EPA, HODOC, NIH, EINECS, IRDB
Contributor: NIST Mass Spectrometry Data Center, 1998.
InChIKey: WQOXQRCZOLPYPM-UHFFFAOYSA-N Non-stereo

RT 5.43 min / RI 892 / 3-Nonene

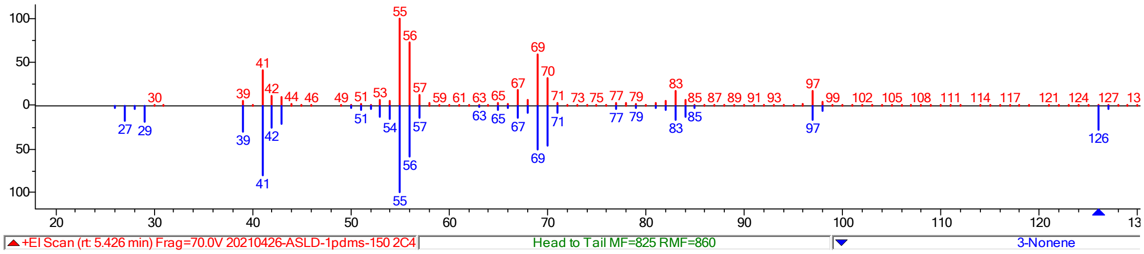

Name: 3-Nonene
Formula: C_9H_{18}
MW: 126 Exact Mass: 126.1408505 CAS#: 20063-77-8 NIST#: 114742 ID#: 22188 DB: mainlib
Other DBs: None
Contributor: NIST Mass Spectrometry Data Center, 1990.
InChIKey: YCBSHDKATAPNIA-FNORWQNLSA-N Non-stereo

RT 5.61 min / RI 903 / Heptanal

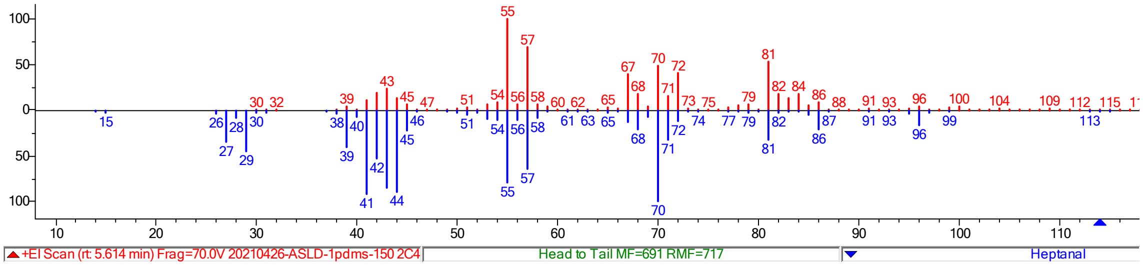

Name: Heptanal
Formula: $C_7H_{14}O$
MW: 114 Exact Mass: 114.104465 CAS#: 111-71-7 NIST#: 341312 ID#: 42683 DB: mainlib

Other DBs: Fine, TSCA, RTECS, HODOC, NIH, EINECS
Contributor: NIST Mass Spectrometry Data Center
InChIKey: FXHGMKSSBGDXIY-UHFFFAOYSA-N   Non-stereo

RT 6.17 min / RI 936 / α-Pinene

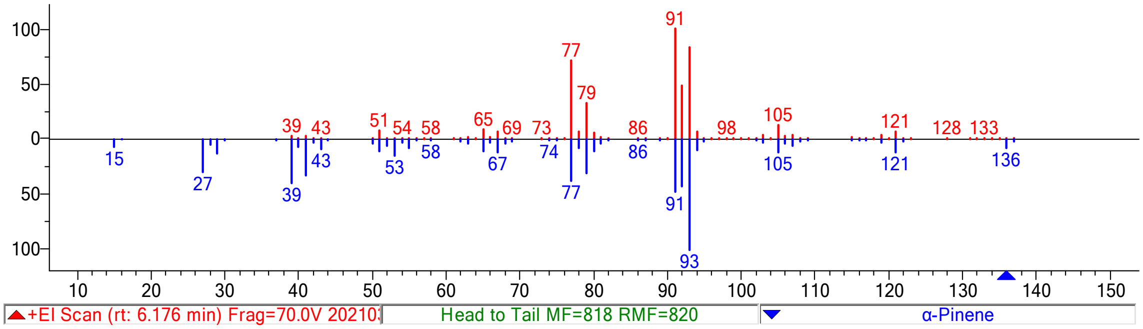

Name: α-Pinene
Formula: $C_{10}H_{16}$
MW: 136 Exact Mass: 136.1252 CAS#: 80-56-8 NIST#: 134072 ID#: 82509 DB: mainlib
Other DBs: TSCA, RTECS, EPA, NIH, EINECS, IRDB
Contributor: NIST Mass Spectrometry Data Center, 1994
InChIKey: GRWFGVWFFZKLTI-UHFFFAOYSA-N   Non-stereo
Related CAS#: 2437-95-8

RT 6.46 min / RI 953 / Camphene

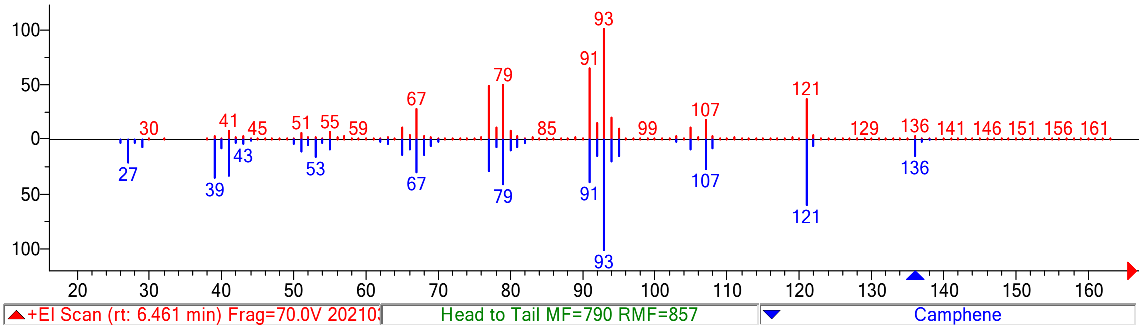

Name: Camphene
Formula: $C_{10}H_{16}$
MW: 136 Exact Mass: 136.1252 CAS#: 79-92-5 NIST#: 114291 ID#: 82854 DB: mainlib
Other DBs: Fine, TSCA, RTECS, EPA, NIH, EINECS, IRDB
Contributor: NIST Mass Spectrometry Data Center, 1990.
InChIKey: CRPUJAZIXJMDBK-UHFFFAOYSA-N   Non-stereo

RT 6.92 min / RI 981 / β-Pinene

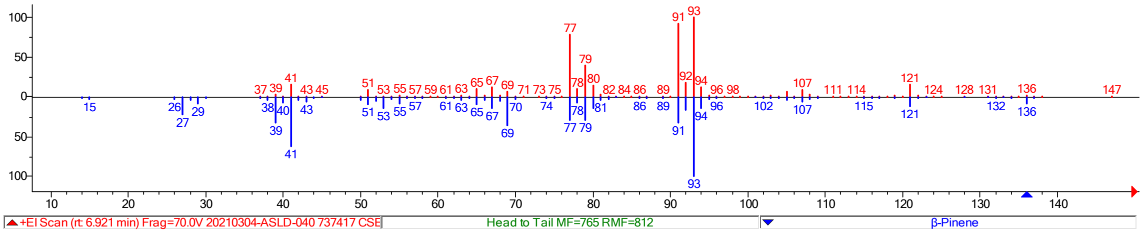

Name: β-Pinene
Formula: $C_{10}H_{16}$

MW: 136 Exact Mass: 136.1252 CAS#: 127-91-3 NIST#: 118895 ID#: 82026 DB: mainlib
Other DBs: TSCA, RTECS, NIH, EINECS, IRDB
Contributor: NIST Mass Spectrometry Data Center, 1990.
InChIKey: WTARULDDTDQWMU-UHFFFAOYSA-N   Non-stereo
Related CAS#: 23089-32-9

RT 7.24 min / RI 1001 / 2-Carene

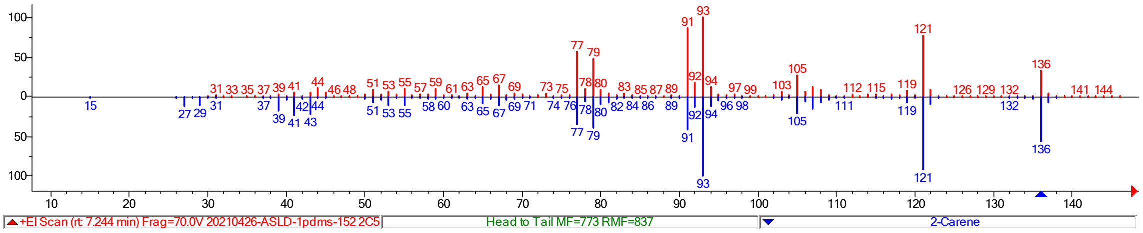

Name: 2-Carene
Formula: $C_{10}H_{16}$
MW: 136 Exact Mass: 136.1252 CAS#: 554-61-0 NIST#: 38898 ID#: 17627 DB: replib
Other DBs: Fine
Contributor: G.VON BUNAU, MAX-PLANCK-INST., MULHEIM-RHUR, GERMANY, FRG
InChIKey: IBVJWOMJGCHRRW-UHFFFAOYSA-N   Non-stereo
Related CAS#: 53702-19-5

RT 7.38 min / RI 1009 / α-Phellandrene

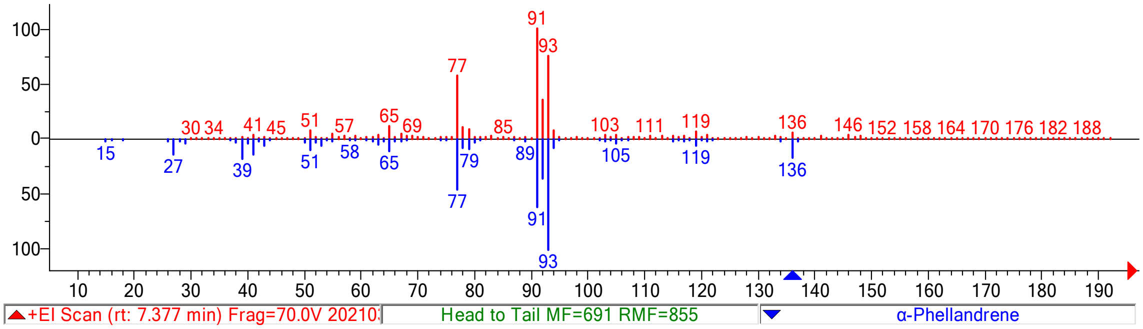

Name: α-Phellandrene
Formula: $C_{10}H_{16}$
MW: 136 Exact Mass: 136.1252 CAS#: 99-83-2 NIST#: 118210 ID#: 17542 DB: replib
Other DBs: TSCA, RTECS, EINECS, IRDB
Contributor: NIST Mass Spectrometry Data Center, 1990.
InChIKey: OGLDWXZKYODSOB-UHFFFAOYSA-N   Non-stereo
Related CAS#: 1330-17-2, 13811-01-3

RT 7.5 min / RI 1019 / α-Terpinene [synonym (1-methyl-4-(1-methylethyl)-1,3-cyclohexadiene)]

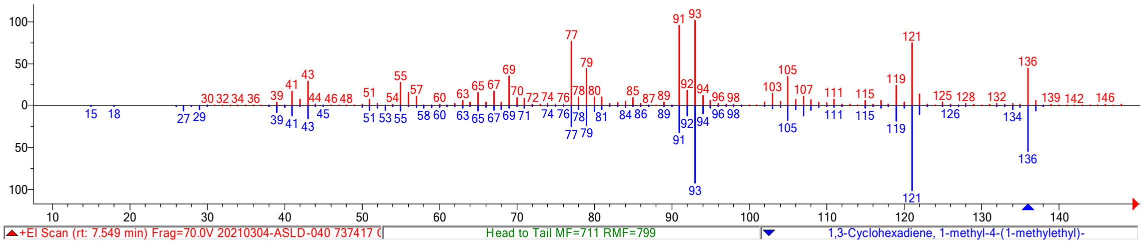

Name: 1,3-Cyclohexadiene, 1-methyl-4-(1-methylethyl)-
Formula: $C_{10}H_{16}$
MW: 136 Exact Mass: 136.1252 CAS#: 99-86-5 NIST#: 237976 ID#: 24065 DB: replib
Other DBs: Fine, TSCA, RTECS, EPA, HODOC, EINECS, IRDB
Contributor: Japan AIST/NIMC Database- Spectrum MS-NW-9574
InChIKey: YHQGMYUVUMAZJR-UHFFFAOYSA-N Non-stereo

RT 7.68 min / RI 1028 / P-Cymene

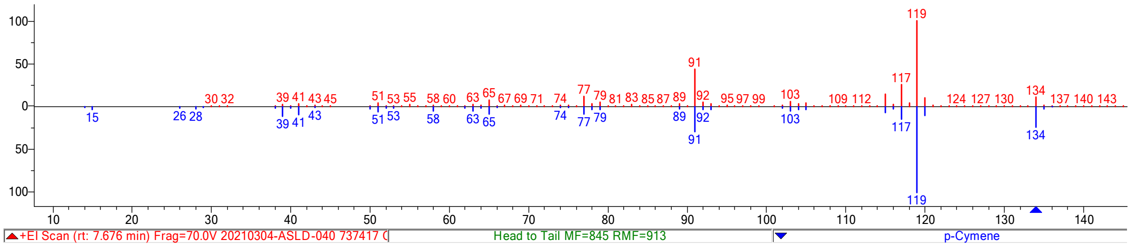

Name: p-Cymene
Formula: $C_{10}H_{14}$
MW: 134 Exact Mass: 134.10955 CAS#: 99-87-6 NIST#: 436742 ID#: 23342 DB: replib
Other DBs: Fine, TSCA, RTECS, EPA, HODOC, NIH, EINECS, IRDB
Contributor: V.A.Korolev, Moscow, Russia
InChIKey: HFPZCAJZSCWRBC-UHFFFAOYSA-N Non-stereo

RT 7.75 min / RI 1032 / D-limonene

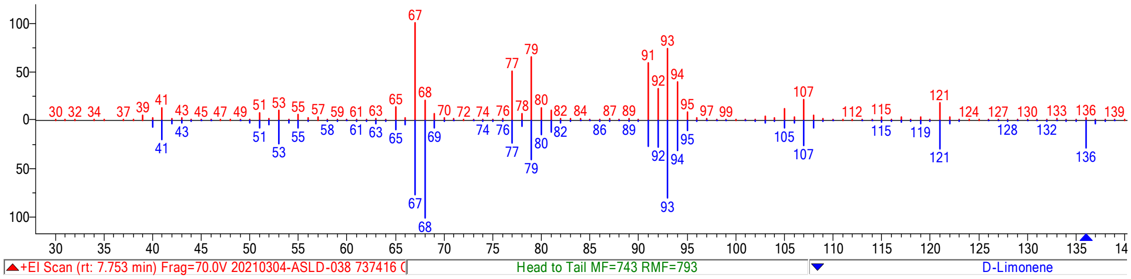

Name: D-Limonene
Formula: $C_{10}H_{16}$
MW: 136 Exact Mass: 136.1252 CAS#: 5989-27-5 NIST#: 36573 ID#: 10180 DB: replib
Other DBs: Fine, TSCA, RTECS, HODOC, EINECS, IRDB
Contributor: R.T.HOLMAN,UNIVERSITY OF MINNESOTA
InChIKey: XMGQYMWWDOXHJM-UHFFFAOYSA-N Non-stereo
Related CAS#: 7705-13-7, 95327-98-3

RT 7.78 min / RI 1034 / β-Phellandrene

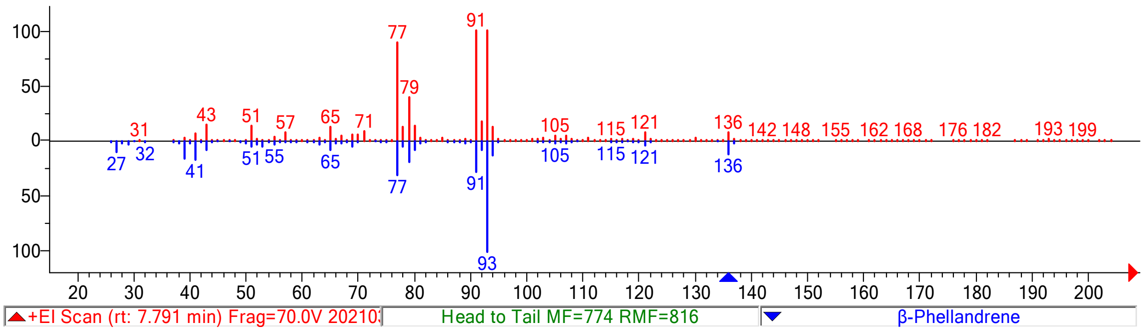

Name: β-Phellandrene
Formula: $C_{10}H_{16}$
MW: 136 Exact Mass: 136.1252 CAS#: 555-10-2 NIST#: 151434 ID#: 17502 DB: replib
Other DBs: TSCA, HODOC, NIH, EINECS
Contributor: Chemical Concepts
InChIKey: LFJQCDVYDGGFCH-UHFFFAOYSA-N Non-stereo

RT 7.82 min / RI 1037 / Benzyl alcohol

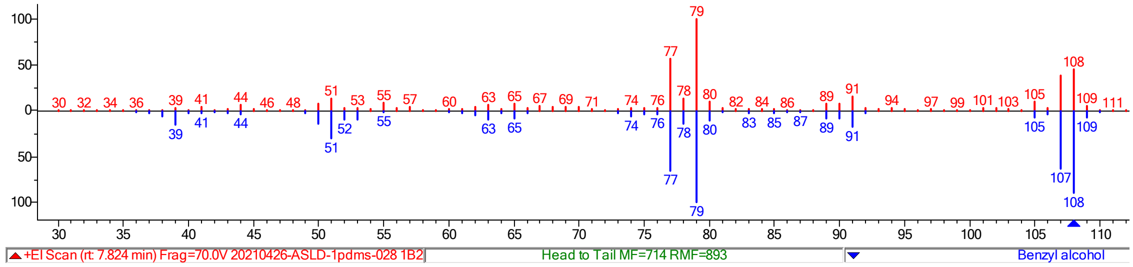

Name: Benzyl alcohol
Formula: C_7H_8O
MW: 108 Exact Mass: 108.0575147 CAS#: 100-51-6 NIST#: 379408 ID#: 14026 DB: replib
Other DBs: Fine, TSCA, RTECS, EPA, USP, HODOC, NIH, EINECS, IRDB
Contributor: Drug Lab
InChIKey: WVDDGKGOMKODPV-UHFFFAOYSA-N Non-stereo
Related CAS#: 185532-71-2, 1336-27-2

RT 8.22 min / RI 1061 / γ-Terpinene

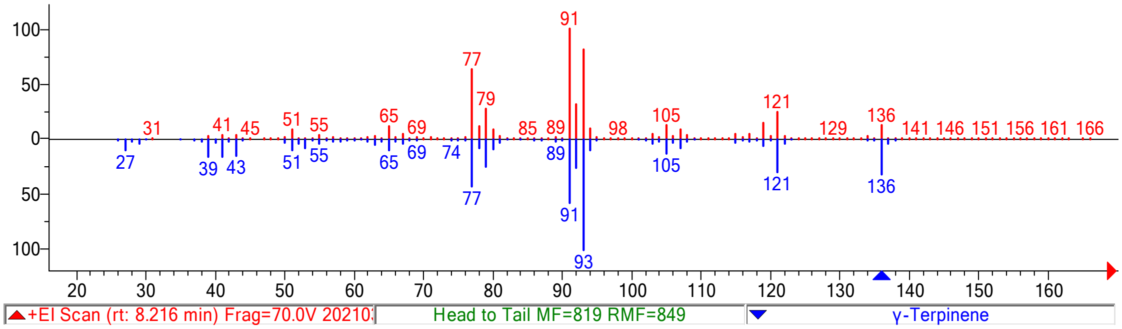

Name: γ-Terpinene
Formula: $C_{10}H_{16}$

[MW:](#) 136 [Exact Mass:](#) 136.1252 [CAS#:](#) 99-85-4 [NIST#:](#) 161219 [ID#:](#) 17538 [DB:](#) replib
[Other DBs:](#) Fine, TSCA, RTECS, HODOC, NIH, EINECS
[Contributor:](#) Chemical Concepts
[InChIKey:](#) YKFLAYDHMOASIY-UHFFFAOYSA-N Non-stereo

RT 9.76 min / RI 1161 / 2-Nonenal

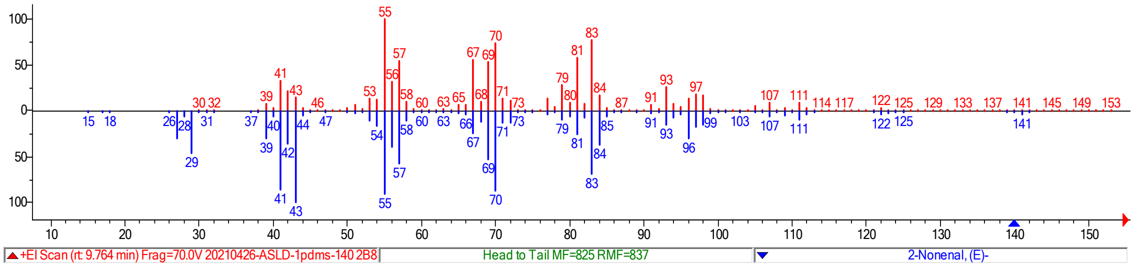

[Name:](#) 2-Nonenal, (E)-
[Formula:](#) $C_9H_{16}O$
[MW:](#) 140 [Exact Mass:](#) 140.120115 [CAS#:](#) 18829-56-6 [NIST#:](#) 238707 [ID#:](#) 2547 [DB:](#) replib
[Other DBs:](#) Fine, TSCA, RTECS, HODOC, EINECS
[Contributor:](#) Japan AIST/NIMC Database- Spectrum MS-NW-8842
[InChIKey:](#) BSAIUMLZVGUGKX-BQYQJAHWSA-N Non-stereo

RT 9.85 min / RI 1166 / 1-(2-hydroxyphenyl)-ethanone

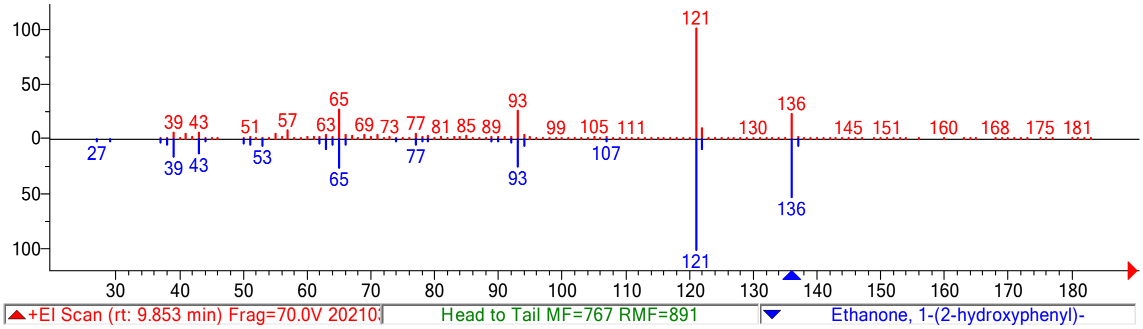

[Name:](#) Ethanone, 1-(2-hydroxyphenyl)-
[Formula:](#) $C_8H_8O_2$
[MW:](#) 136 [Exact Mass:](#) 136.052429 [CAS#:](#) 118-93-4 [NIST#:](#) 288831 [ID#:](#) 24213 [DB:](#) replib
[Other DBs:](#) Fine, TSCA, RTECS, EPA, HODOC, NIH, EINECS
[Contributor:](#) James Little,Eastman Chem.Co.,Kingsport,TN
[InChIKey:](#) JECYUBVRTQDVAT-UHFFFAOYSA-N Non-stereo

RT 10.34 min / RI 1199 / Methyl salicylate

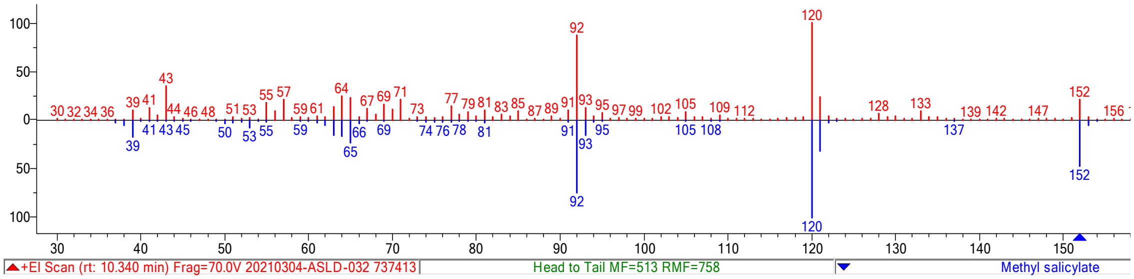

Name: Methyl salicylate
Formula: $C_8H_8O_3$
MW: 152 Exact Mass: 152.047344 CAS#: 119-36-8 NIST#: 312943 ID#: 23704 DB: replib
Other DBs: Fine, TSCA, RTECS, USP, HODOC, NIH, EINECS, IRDB
Contributor: Dr. P.K. Shah, NYC Police Laboratory, NY
InChIKey: OSWPMRLSEDHDFF-UHFFFAOYSA-N   Non-stereo
Related CAS#: 8022-86-4, 648434-07-5, 8024-54-2

Table S2. *Spodoptera exigua* artificial diet (per 1L)

**Ingredients**	**Grams/Litre**
Agar	28 g/l
Cornflower, polenta	160 g/l
Beer-yeast	50 g/l
Wheat-germs	50 g/l
Sorbic acid	2 g/l
Nipagin (methyl-4-hydroxybenzoate)	1.6 g/l
Ascorbic acid (vitamin C)	8 g/l
Streptomycin	0.1 g/l

Table S3. Parameters used for data processing using MZmine 2.53 (ADAP)

Process Step	Parameter [Unit]	Value
Mass Detection (centroid)	Retention time range [min]	1-25.5
	Noise Level	1.00E+03
Chromatogram Building (ADAP)	Minimum group size [unit]	5
	Group intensity threshold	200
	m/z Tolerance [mz / ppm]	0.02 / 5
Peak Deconvolution (Wavelets ADAP)	S/N threshold	3
	Minimum feature height	1000
	Coefficient/area threshold	200
	Peak duration range [min]	0.0-1.50
	RT wavelet range [min]	0-0.10
Spectral Deconvolution (Hierarchical clustering ADAP)	Minimum cluster distance	0.01
	Minimum cluster size	1
	Minimum cluster intensity	10000
	Minimum edge-to-height ratio	0.3
	Minimum delta-to-height ratio	0.2
	Minimum sharpness	10
	Shape similarity tolerance	18
Alignment (ADAP aligner GC)	Minimum confidence	0.1
	Retention time tolerance (min)	0.01
	m/z Tolerance [mz/ppm]	0.01/ 5.0
	Score threshold	0.75
	Score weight	0.1

FigureS2. Comparison of tomato root biomass between the treatments (CSE_{PIM}, SE_{PIM}, CSE_{MON}, SE_{MON}). Root biomass is not statistically different between the treatments according to ANOVA test (p-value = 0.599).

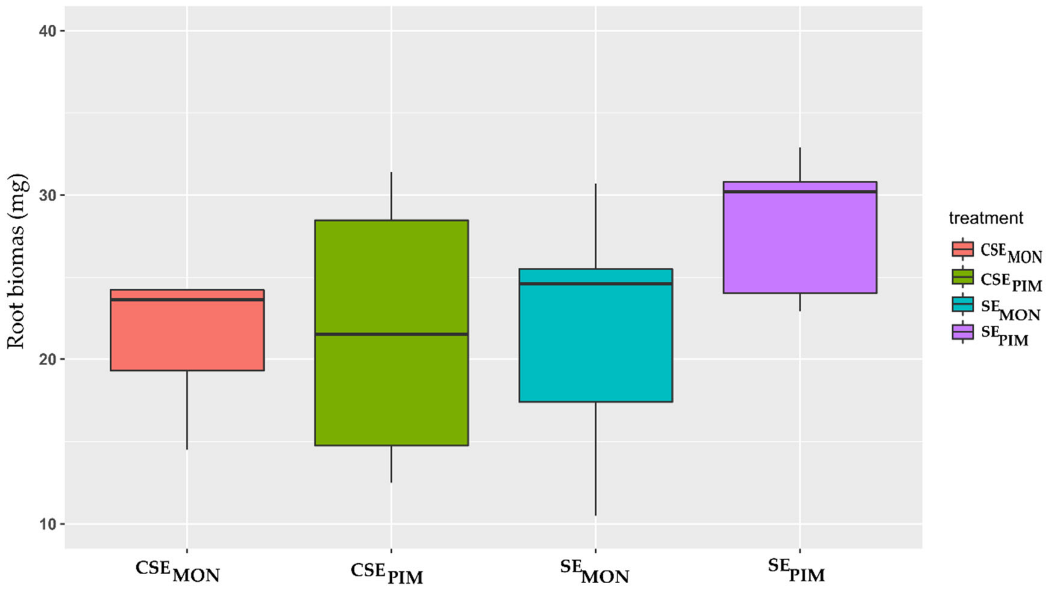

FigureS3. A scatter plot visualizing the correlation between peak intensity of model compound α-pinene and tomato root biomass (mg, dry weight). Pearson correlation indicated no correlation between the peak intensity model with root biomass (p-value = 0.5961).

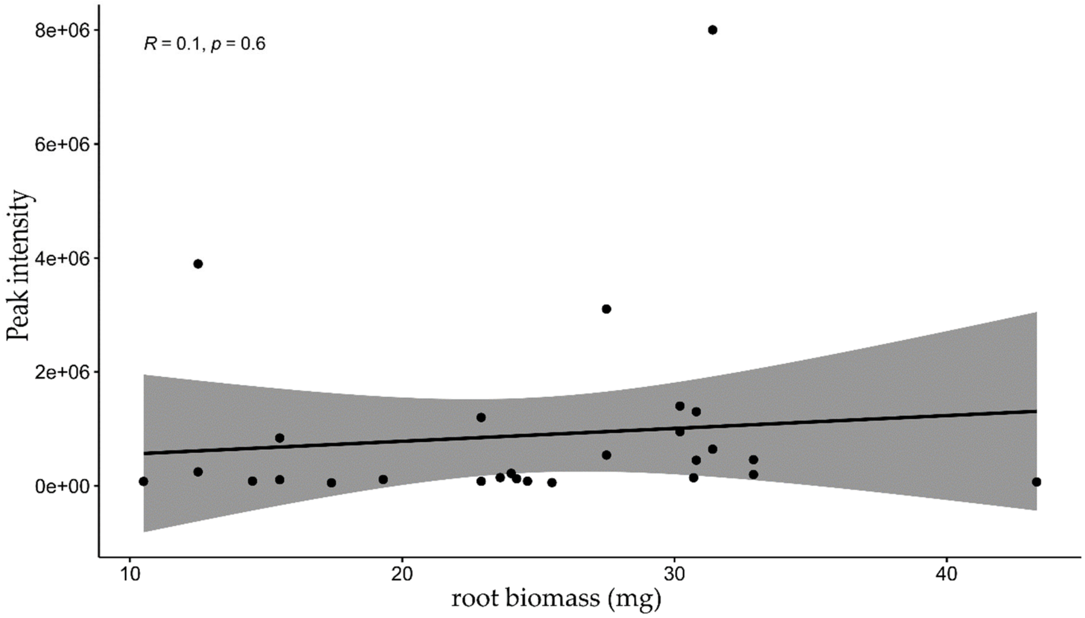

Supplement: Supplementary file 1 [file molecules-27-01612-s001.zip › molecules-1546322-supplementary.pdf]
